# MR Imaging–derived Oxygen-Hemoglobin Dissociation Curves and Fetal-Placental Oxygen-Hemoglobin Affinities

**DOI:** 10.1148/radiol.2015150721

**Published:** 2016-01-14

**Authors:** Reut Avni, Ofra Golani, Ayelet Akselrod-Ballin, Yonni Cohen, Inbal Biton, Joel R. Garbow, Michal Neeman

**Affiliations:** From the Departments of Biological Regulation (R.A., A.A.B., Y.C., M.N.), Biological Services (O.G.), and Veterinary Resources (I.B.), Weizmann Institute of Science, Rehovot 76100, Israel; and Biomedical Magnetic Resonance Laboratory, Mallinckrodt Institute of Radiology, Washington University School of Medicine, St Louis, Mo (J.R.G.).

## Abstract

**Purpose:**

To generate magnetic resonance (MR) imaging–derived, oxygen-hemoglobin dissociation curves and to map fetal-placental oxygen-hemoglobin affinity in pregnant mice noninvasively by combining blood oxygen level–dependent (BOLD) T2* and oxygen-weighted T1 contrast mechanisms under different respiration challenges.

**Materials and Methods:**

All procedures were approved by the Weizmann Institutional Animal Care and Use Committee. Pregnant mice were analyzed with MR imaging at 9.4 T on embryonic days 14.5 (eight dams and 58 fetuses; imprinting control region ICR strain) and 17.5 (21 dams and 158 fetuses) under respiration challenges ranging from hyperoxia to hypoxia (10 levels of oxygenation, 100%–10%; total imaging time, 100 minutes). A shorter protocol with normoxia to hyperoxia was also performed (five levels of oxygenation, 20%–100%; total imaging time, 60 minutes). Fast spin-echo anatomic images were obtained, followed by sequential acquisition of three-dimensional gradient-echo T2*- and T1-weighted images. Automated registration was applied to align regions of interest of the entire placenta, fetal liver, and maternal liver. Results were compared by using a two-tailed unpaired Student *t* test. R1 and R2* values were derived for each tissue. MR imaging–based oxygen-hemoglobin dissociation curves were constructed by nonlinear least square fitting of 1 minus the change in R2*divided by R2*at baseline as a function of R1 to a sigmoid-shaped curve. The apparent P50 (oxygen tension at which hemoglobin is 50% saturated) value was derived from the curves, calculated as the R1 scaled value (*x*) at which the change in R2* divided by R2*at baseline scaled (*y*) equals 0.5.

**Results:**

The apparent P50 values were significantly lower in fetal liver than in maternal liver for both gestation stages (day 14.5: 21% ± 5 [*P* = .04] and day 17.5: 41% ± 7 [*P* < .0001]). The placenta showed a reduction of 18% ± 4 in mean apparent P50 values from day 14.5 to day 17.5 (*P* = .003). Reproduction of the MR imaging–based oxygen-hemoglobin dissociation curves with a shorter protocol that excluded the hypoxic periods was demonstrated.

**Conclusion:**

MR imaging–based oxygen-hemoglobin dissociation curves and oxygen-hemoglobin affinity information were derived for pregnant mice by using 9.4-T MR imaging, which suggests a potential to overcome the need for direct sampling of fetal or maternal blood.

*Online supplemental material is available for this article.*

## Abstract (synopsis)

The authors of this study present a noninvasive approach for obtaining MR imaging–based oxygen-hemoglobin dissociation curves and for deriving oxygen tension values at which hemoglobin is 50% saturated and maps for the placenta and fetus in pregnant mice.

## Introduction

Oxygen transport between the placenta and the fetus is one of several key functions performed by the placenta, and compromised oxygen transport can have severe negative consequences for fetal growth and development (1,2). Placental oxygen transport depends on several parameters, including the rates of blood flow through the maternal and fetal placental circulation, hemoglobin concentration, the mean oxygen pressure gradient between maternal and fetal blood, oxygen capacity, and the oxygen affinity of fetal and maternal blood (3,4).

The oxygen-hemoglobin dissociation curve characterizes the sigmoid relationship among the saturation of hemoglobin, oxygen saturation (So_2_oxygen saturation), and the partial pressure of oxygen (Po_2_partial pressure of oxygen). The predominant hemoglobin of the fetus for most of the gestation period is fetal hemoglobin, which has a greater oxygen affinity than does adult hemoglobin. This increased affinity permits adequate delivery of oxygen to fetal tissue at the lower oxygen levels found in the fetus (3–8).

Oxygen affinity can be measured as the Po_2_partial pressure of oxygen level at which hemoglobin is 50% saturated with oxygen (P50). To obtain P50 values, Po_2_partial pressure of oxygen and So_2_oxygen saturation can be determined ex vivo from arterial and venous blood samples (9). However, during pregnancy, extraction of blood samples from the fetal umbilical cord is a highly invasive procedure, involving a great risk to the fetus (10). Performing such studies in a mouse model is technically challenging due to the small diameter of fetal blood vessels (11). Noninvasive imaging methods that can probe fetal-placental oxygen affinity can provide important insights into the placental oxygen transfer mechanism and contribute to our understanding of the risk factors that may lead to breakdown in this function.

Functional magnetic resonance (MR) imaging techniques such as blood oxygen level dependent (BOLDblood oxygen level dependent) and T1 contrast can provide noninvasive information about the oxygen environment. BOLDblood oxygen level dependent contrast MR imaging is a tool for studying the oxygenation of blood by using endogenous hemoglobin as a reporter molecule (12,13). Changes in T2* during a respiration challenge reflect changes in deoxyhemoglobin content, and, thus, are affected by So_2_oxygen saturation (14–16). Studies in sheep have shown that changes in fetal BOLDblood oxygen level dependent MR imaging signal intensity are closely related to changes in fetal oxygenation estimated with fetal arterial hemoglobin saturation (17,18), and with fluorescent oxygen sensors inserted into the fetal liver (19). BOLDblood oxygen level dependent MR imaging has been applied in pregnant mice under a hypoxic respiration challenge (20) in a maternal model of chronic fetal asphyxia (21) and in a rat model of intrauterine growth restriction (22,23). BOLDblood oxygen level dependent MR imaging also has been applied in humans in normal (24,25) and intrauterine growth restriction–compromised pregnancies (26), demonstrating changes in oxygenation in the placenta and in different fetal organs under maternal oxygen challenge. While BOLDblood oxygen level dependent MR imaging reports on hemoglobin saturation, T1 contrast under oxygen challenge is affected mainly by molecular oxygen, which shortens T1 relaxation by means of dipolar interactions. Therefore, oxygen-induced changes in T1 reflect mainly Po_2_partial pressure of oxygen levels (16,27–32). BOLDblood oxygen level dependent MR imaging has been applied with oxygen-enhanced T1 contrast to investigate the placental oxygen environment throughout a range of gestation times (33).

The purpose of this study was to generate MR imaging–derived oxygen-hemoglobin dissociation curves and to map fetal-placental oxygen-hemoglobin affinity in pregnant mice noninvasively by combining BOLDblood oxygen level dependent T2* and oxygen-weighted T1 contrast mechanisms under different respiration challenges.

## Materials and Methods

### Animals

All experiments were approved by the Weizmann Institutional Animal Care and Use Committee. Female imprinting control region pregnant mice (Harlan Laboratories, Rehovot, Israel) were analyzed under different respiration challenges: *(a)* eight mice (58 fetuses and placentas) at embryonic day 14.5 and 10 mice at embryonic day 17.5 (89 fetuses and placentas) of pregnancy underwent a hyperoxia-to-hypoxia challenge, *(b)* six mice (37 fetuses and placentas) underwent a normoxia-to-hyperoxia challenge at day 17.5, and *(c)* five control group mice (32 fetuses and placentas) were exposed to a constant oxygen level of 100% at day 17.5. The group size of at least five mice was based on a power calculation with estimated changes in signal values of 10% and a standard deviation of 10% for a power of 0.8 and an α of .05. All animals were euthanized at the end of the imaging series.

### In Vivo MR Imaging Studies

MR imaging examinations were performed at 9.4 T with an MR spectrometer (BioSpec 94/20 USR; Bruker, Karlsruhe, Germany) equipped with a gradient-coil system capable of producing pulsed gradients of up to 0.004 T per centimeter in each of three orthogonal directions. A quadrature volume coil with a 72-mm inner diameter and a homogeneous radiofrequency field of 100 mm along the axis of the magnetic field was used for both transmission and reception. During MR imaging, dams were anesthetized with isoflurane (3% for induction, 1%–2% for maintenance; Abbott Laboratories, West Berkshire, England) mixed with 1 L/min of oxygen and nitrogen, delivered through a nasal mask. Once anesthetized, the animals were placed in a supine position in a head holder to ensure reproducible positioning inside the magnet. Respiration rate was monitored by using an monitoring and gating system (Model 1025; SA Instruments, Stony Brook, NY) and maintained throughout the experimental period at approximately 50 breaths per minute by adjusting the isoflurane level. The oxygenation status was monitored by using a pulse oximeter probe (SA Instruments) placed on the mouse’s tail. Body temperature was maintained at approximately 37°C with a circulating water heating system, placed under the animal bed.

### MR Imaging Data Acquisition

Oxygen transfer in pregnant mice was monitored with MR imaging (Figs 1, 2). The hyperoxia-to-hypoxia challenge was applied by using 100%, 80%, 70%, 60%, 50%, 40%, 30%, 20%, 15%, and 10% oxygen. At each oxygen phase, the nitrogen level was adjusted to maintain a constant flow of inhaled gas, and anatomic data were acquired by using fast spin-echo rapid acquisition with refocused echoes, followed by sequential acquisition of three-dimensional gradient-echo T2*-weighted BOLDblood oxygen level dependent contrast and three-dimensional gradient-echo T1-weighted sequences, resulting in approximately 8 minutes of imaging time per oxygen phase, with a total of 100 minutes of imaging time.

**Figure 1a:**
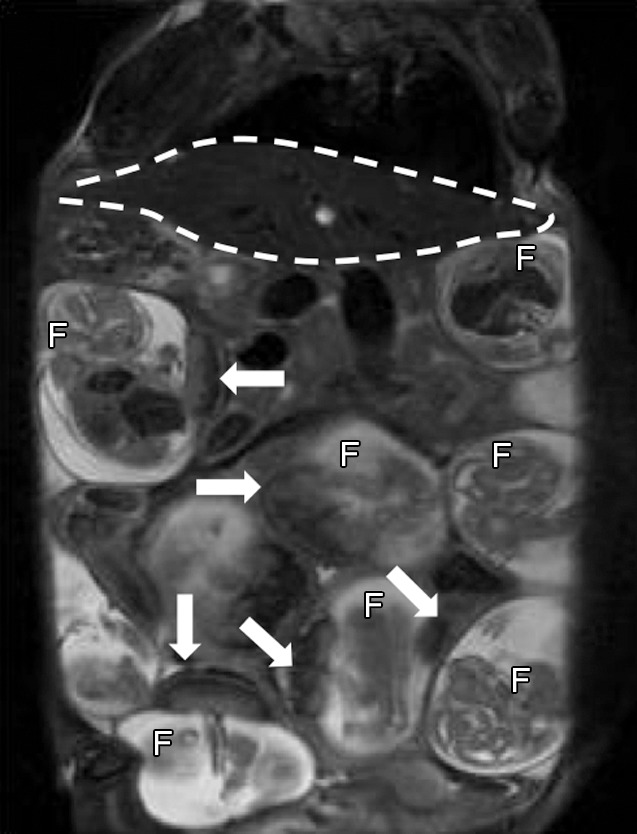
Anatomic T2-weighted fast spin-echo rapid acquisition with refocused echoes images of imprinting control region pregnant mouse at embryonic day 17.5 show several fetuses *(F)* and their placentas (arrows). Regions of interest (dotted lines) were drawn on the basis of anatomic images for each type of tissue: **(a)** Region of interest placement in maternal liver (white dotted line) and **(b)** region of interest placements in the placenta (black dotted line) and fetal liver (white dotted line).

**Figure 1b:**
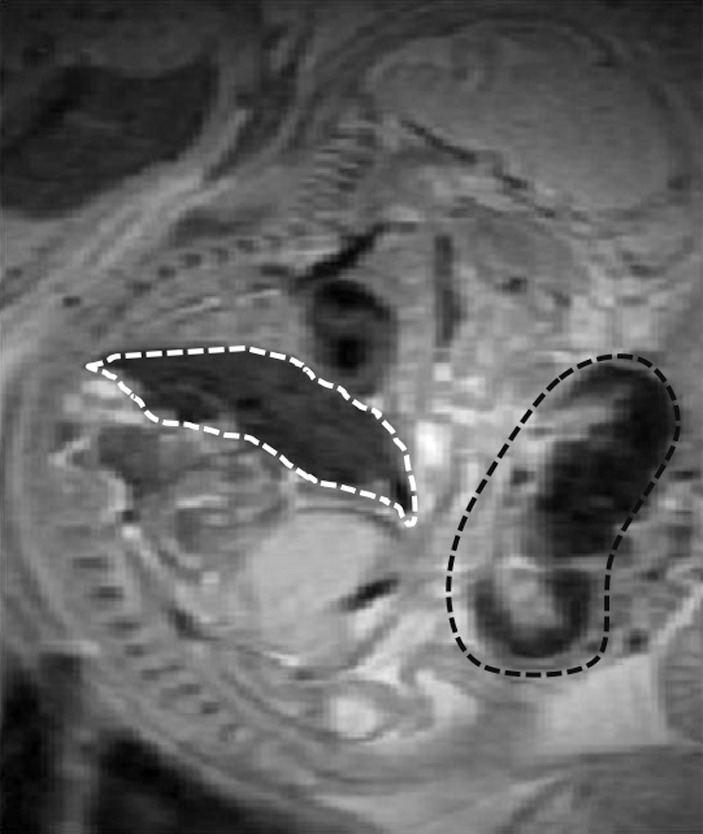
Anatomic T2-weighted fast spin-echo rapid acquisition with refocused echoes images of imprinting control region pregnant mouse at embryonic day 17.5 show several fetuses *(F)* and their placentas (arrows). Regions of interest (dotted lines) were drawn on the basis of anatomic images for each type of tissue: **(a)** Region of interest placement in maternal liver (white dotted line) and **(b)** region of interest placements in the placenta (black dotted line) and fetal liver (white dotted line).

**Figure 2:**
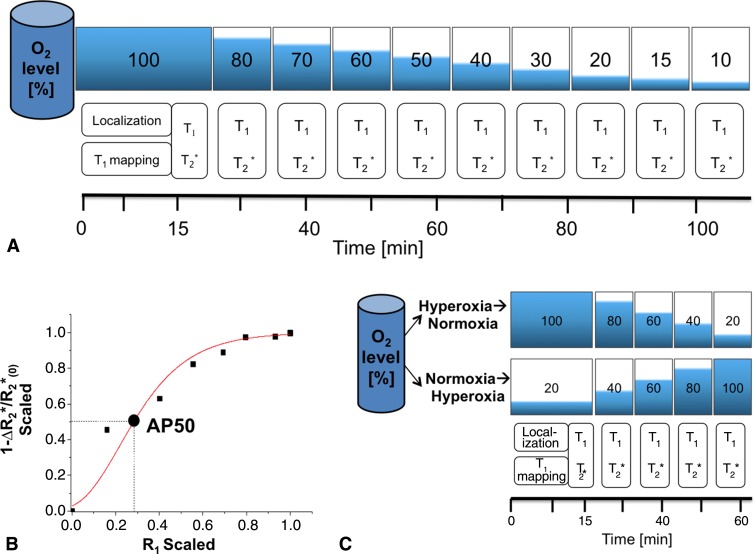
Illustrations and graph show MR imaging analysis of apparent hemoglobin dissociation (AP50). *A*, Pregnant mice were analyzed by using hyperoxia-to-hypoxia respiration challenge with gradual decrease in fractional inhaled oxygen level from 100% to 10%. T1 mapping was performed at the initial phase, three-dimensional gradient-echo T2*-weighted and T1-weighted images were acquired sequentially at each oxygen phase. Each phase was maintained for approximately 8 minutes, resulting in approximately 100 minutes of total imaging time per mouse. *B*, Example of MR imaging-based oxygen hemoglobin dissociation curve (squares and circle) fitted to Hill function (red line) to extract MR imaging–based AP50 value. *C*, Illustration shows minimal configuration for mapping AP50. Hyperoxia-to-hypoxia data were reanalyzed with subset of inhaled oxygen phases, excluding hypoxic period (hyperoxia-to-normoxia), and an additional experiment was performed by using a normoxia-to-hyperoxia respiration challenge.

A rapid acquisition with refocused echoes sequence was used to visualize fetal-placental units (Fig 1) with the following parameters: repetition time msec/effective echo time msec 4300/21; rapid acquisition with refocused echoes factor, eight; section thickness, 0.5 mm; number of sections, 64; field of view, 5.5 × 5.5 cm^2^; matrix, 256 × 128, zero filled to 256 × 256; averages, two (pixel size after zero filling, 0.214 mm^2^).

The initial longitudinal relaxation rate (R1 = 1/T1) was measured by using a series of variable flip angle three-dimensional gradient-echo images with the following parameters: 20/3.0; pulse flip angle, 5°, 15°, 30°, 50°, and 70°; field of view, 5.5 × 5.5 × 3.2 cm^3^; matrix, 128 × 128 × 64, zero filled to 256 × 256 × 64; averages, two. T1- and T2*-weighted images were acquired as above with a flip angle of 15° and echo time of 3.0 msec and 8.5 msec, respectively.

### MR Imaging Data Analysis

*Anatomic evaluation.—*MR imaging data analysis was performed by an MR imaging physicist (R.A., with 5 years of experience in mouse fetal and placental imaging). We evaluated three highly vascularized organs: fetal liver, placenta, and maternal liver. Regions of interest were manually drawn for the entire organs, selected on the basis of the rapid acquisition with refocused echoes anatomic sequence. To correct for individual motion of embryos between consecutive sequences, an automated algorithm for serial alignment was applied (34). Multiple regions of interest were selected in several sections and each volume sequence was individually registered on the basis of intensity and geometric features derived from the anatomic images. All further maps were calculated from the registered images.

Construction of T1 and T2* *maps.—*R1 maps were derived from the variable flip-angle data by means of nonlinear fitting with the following equation:
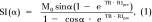
where SI is the signal intensity as a function of the pulse flip angle, α, and the pre-exponent term, TR is the repetition time, R1_pre_ is the relaxation rate at the baseline level, and *M*_0_ includes contributions from both spin density and T2 relaxation.

R1 values at each oxygen phase (R1_t_) were extrapolated from the baseline by using the T1-weighted signal intensity values, SI_0_ and SI_t_, respectively.
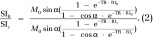
R1 values were corrected for imperfections in flip-angle distribution that may lead to errors (Equation E1, Appendix E1 [online]).

R2* maps were derived from dual-echo images acquired with two different echo times (8.5 and 3 msec). The ratio of the two signal intensity values, SI_TE1_ and SI_TE2_, can be expressed as:
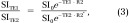
where SI_0_ is the baseline signal intensity, TE1 and TE2 are the first and second echo times, respectively, and T2* is the transverse relaxation time constant. This equation can be rearranged to solve for R2*:
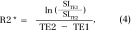
where ln is the natural logarithm.

*Construction of fetal-placental oxygen-hemoglobin dissociation curves.—*Derivation of apparent P50 (hereafter, AP50) hemoglobin dissociation, or oxygen tension at which hemoglobin is 50% saturated, is based on the linear dependence of R1 on Po_2_partial pressure of oxygen (29,32) and that of 1 minus the change in R2* divided by R2* at baseline (1 − ΔR2*/R2*_0_) on So_2_oxygen saturation, where ΔR2* is the difference between R2* value at a given deoxyhemoglobin level (R2*_Hb_) and the baseline level (R2*_0_), measured at 100% oxygen:
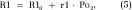

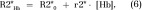

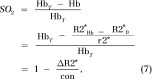
In these equations, r1 is the molecular oxygen-induced relaxivity, r2* is the deoxyhemoglobin-induced relaxivity, *con* is a constant, ΔR2* is the change in R2*, and Hb and Hb_*T*_ are the concentrations of deoxyhemoglobin and total hemoglobin, respectively. The oxygen hemoglobin dissociation curve is described by the Hill function (35,36). In general, So_2_oxygen saturation ranges between 0 and 1, whereas Po_2_partial pressure of oxygen levels are usually measured in units of millimeters of mercury. However, the MR imaging−based parameters may vary, depending on the initial R2* and R1 values. Therefore, a linear transformation (trans) was performed, scaling all *x* (R1) and *y* (1 − ΔR2*/R2*_0_) values to span the range from 0 to 1 (Eq 9):
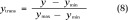

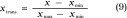
The scaled curves were then fitted to the sigmoid-shaped Hill function by using a least-squares method (Matlab; Math Works, Natick, Mass):
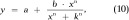
where *a*, *b*, and *k* are constants and n is the slope of the sigmoid.

Finally, to construct MR imaging–based oxygen-hemoglobin dissociation curves, scaled 1 − ΔR2*/R2*_0_ values were plotted as a function of scaled R1 values for each oxygen phase with an overall *R*-squared value of 0.84 ± 0.13. MR imaging–based AP50 values equivalent to standard AP50 values were then derived from the fitted curves, calculated as the R1 scaled value (*x*) at which 1 − ΔR2*/R2*_0_ scaled (*y*) equals 0.5. Pixel-by-pixel analysis was used to generate AP50 maps.

### Minimal (Translatable) Configuration for Mapping AP50

To test the applicability of this experimental setup in a clinically relevant setting, we applied two approaches: *(a)* the hyperoxia-to-hypoxia respiration challenge was reanalyzed, excluding the hypoxic challenge, and with a subset of the inhaled oxygen phases: 100%, 80%, 60%, 40% and 20% oxygen (hyperoxia-to-normoxia respiration challenge) and *(b)* additional experimental data were acquired for a normoxia-to-hyperoxia respiration challenge, with the following oxygen phases: 20%, 40%, 60%, 80%, and 100% oxygen, resulting in total of 60 minutes of imaging time in pregnant mice at day 17.5 (six mice, 37 fetuses and placentas) (Fig 2, *C*). For each of these approaches, mean AP50 values were derived for fetal liver, placenta, and maternal liver and compared with the values obtained by using the full hyperoxia-to-hypoxia procedure.

### Statistical Analysis

Statistical analysis was performed by using software (Statistica; StatSoft, Tulsa, Okla). Data were tested for their normal distribution by means of the Shapiro-Wilks test. An independent, two-tailed, unpaired Student *t* test was applied for analysis of the significance of the MR imaging data, including the comparison between gestation stages. The data were considered to indicate a significant difference when *P* values were less than .05.

## Results

### Anatomic Fetal-Placental MR Imaging

Serial coronal T2-weighted images allowed clear identification and localization of all fetuses and the maternal liver (Fig 1a). By using a fast spin-echo sequence, we were able to easily detect both the placenta and fetal liver, with high contrast and resolution (Fig 1). The average size ± standard deviationof the region of interest for the maternal liver (401.1 mm^2^ ± 95.0) was significantly larger than the other regions of interest (94.8 mm^2^ ± 18.2 for the placenta and 60.6 mm^2^ ± 22.9 for fetal liver; all *P* values < .0001).

### Gradual Maternal Respiration Challenge Corresponds with R1 and R2* Changes in Placenta, Fetal Liver, and Maternal Liver

**Figure 3:**
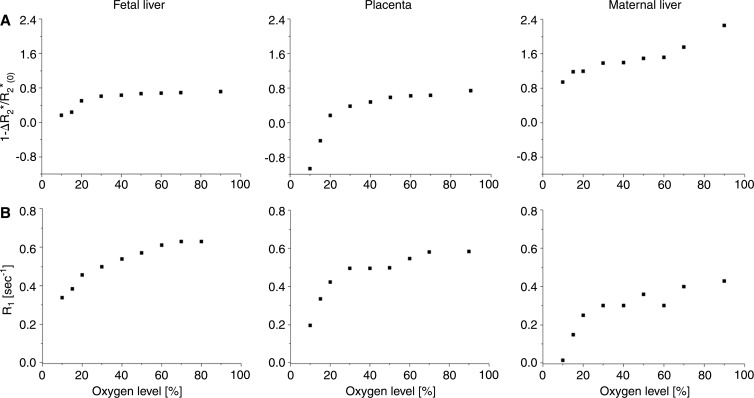
Graphs show that gradual hyperoxia-to-hypoxia respiration challenge results in 1 − ΔR2*/R2_(0)_* and R1 decreases in fetal liver, placenta, and maternal liver. Representative examples of fetal liver (left), placenta (middle), and maternal liver (right) obtained in pregnant mice on day 17.5. *A*, 1 − ΔR2*/R2_(0)_* and, *B*, R1 values are plotted as a function of inhaled oxygen level.

In the hyperoxia-to-hypoxia respiration challenge, both R1 and 1 − ΔR2*/R2*_0_ values decreased gradually, corresponding to the decrease in the inhaled oxygen level, where the most prominent changes were observed for the hypoxic period (< 20% oxygen, Fig 3). Control mice exposed to a constant oxygen level (100%) showed only small variations in R1 and 1 − ΔR2*/R2*_0_ values in all tissues throughout time (Fig E1 [online]).

### Deriving MR Imaging-based AP50 Values

**Figure 4:**
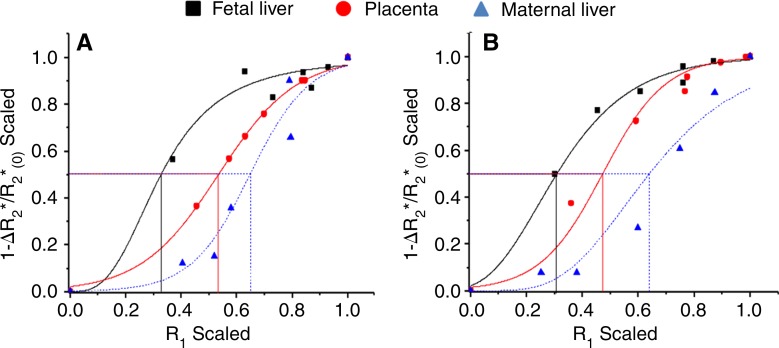
Graphs show representative examples of MR imaging–based oxygen-hemoglobin dissociation curves and derived AP50 values in fetal liver (black), placenta (red), and maternal liver (blue) demonstrating expected sigmoid-shaped function, with shift to left between fetal and adult tissue on days, *A*, 14.5 and, *B*, 17.5.

The experimental results of the derived oxygen dissociation curve were consistent with the expected sigmoid-shaped curve, characterizing the relationship between Po_2_partial pressure of oxygen and the saturation of hemoglobin. The expected left shift for fetal MR imaging–based oxygen-hemoglobin dissociation curves was observed (Fig 4).

**Figure 5:**
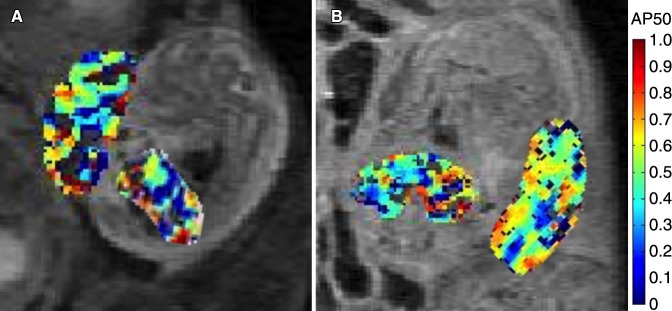
Representative AP50 maps inside the placenta and fetal liver on days, *A*, 14.5 and, *B*, 17.5 show distribution and variability of AP50 values.

On embryonic day 14.5, fetal liver mean AP50 was significantly lower than that of maternal liver (Table 1, Fig E2 [online]). Placental mean AP50 was significantly larger than fetal liver. On day 17.5, placental and fetal liver mean AP50 values were significantly lower than that of maternal liver (*P* < .0001; Table 1, Fig E2 [online]). No significant changes were seen in mean AP50 values from day 14.5 to day 17.5 for either fetal or maternal liver. However, the AP50 of the placenta displayed a significant reduction from day 14.5 to day 17.5 (Table 1). AP50 maps were generated in each type of tissue, showing the spatial distribution of the oxygen-hemoglobin dissociation (Fig 5).

**Table 1:**
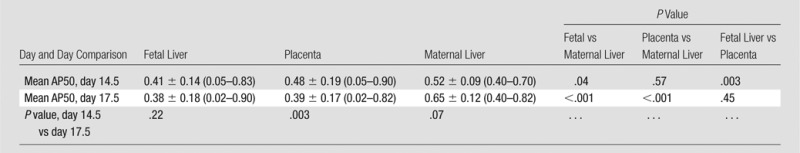
Mean AP50 Values in Fetal Liver, Placenta, and Maternal Liver on Embryonic Days 14.5 and 17.5

Note.—Unless otherwise indicated, data are means ± standard deviation, with the range in parentheses.

### Minimal (Translatable) Configuration for Mapping AP50

**Figure 6:**
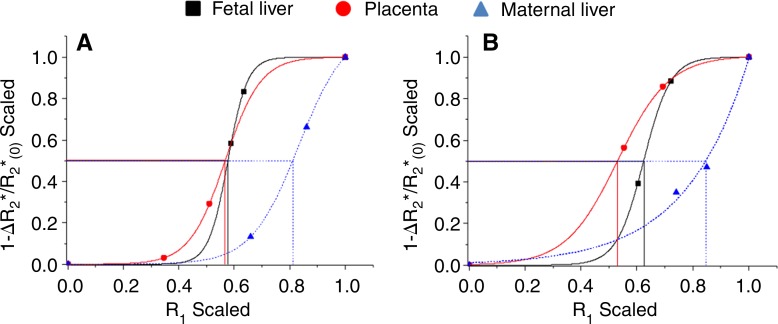
Graphs show analysis of AP50. Representative examples of MR imaging-based, oxygen-hemoglobin dissociation curves and derived AP50 values with limited normoxia-to-hyperoxia protocol in fetal liver (black), placenta (red), and maternal liver (blue) obtained by using, *A*, hyperoxia-to-normoxia or, *B*, normoxia-to-hyperoxia protocols.

A subset of the hyperoxia-to-hypoxia data were reanalyzed excluding hypoxia (hyperoxia-to-normoxia), and the additional experiment with a minimal normoxia-to-hyperoxia respiration challenge exhibited the expected sigmoid-shaped function and the characteristic left shift between fetal and adult tissue (Fig 6). Mean AP50 values were not significantly different between the two minimal protocols for all tissue types (all *P* > .15), but showed small differences for the placenta and fetal liver compared with the results of the full hyperoxia-to-hypoxia protocol (Table 2).

**Table 2:**
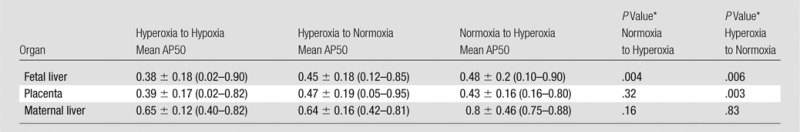
Mean AP50 Values in Fetal Liver, Placenta, and Maternal Liver at Embryonic Day 17.5 by Protocol

Note.—Unless otherwise indicated, data are means ± standard deviation, with the range in parentheses.

*All *P* values shown were calculated by using an independent *t* test relative to the full hyperoxia-to-hypoxia protocol.

## Discussion

In this study, we report on an MR imaging approach for assessing maternal-fetal oxygen transport across the placenta and for mapping fetal and maternal hemoglobin dissociation curves noninvasively. By varying maternal alveolar Po_2_partial pressure of oxygen and So_2_oxygen saturation levels with a respiration challenge, we observed a nonlinear relationship between the inhaled oxygen and MR imaging–derived R1 and R2*. Such a relationship can result from the nonlinear dependence of hemoglobin saturation on oxygen and can affect metabolic rates, oxygen consumption, pH (and hemoglobin-oxygen binding), heart rate, and vessel diameter. Assuming a linear relationship between R1 and Po_2_partial pressure of oxygen and R2* and So_2_oxygen saturation, we derived AP50 values and maps at two gestational stages during placental development in the mice: embryonic day 14.5 (3 days after the formation of the placenta) and embryonic day 17.5 (near term). Although a substantial response of R1 and R2* to the inhaled gas challenges was essential for this analysis, it does not depend on the exact relationship between inhaled oxygen and Po_2_partial pressure of oxygen, but only on linear or approximately linear correlation between R1 and Po_2_partial pressure of oxygen and similarly between R2* and So_2_oxygen saturation. In the mouse model, P50 increased from 29 mm Hg in the fetus to 41 mm Hg at 2 weeks after birth, a mean difference of 29% between the adult and fetus (37). As expected, AP50 values were significantly lower in fetal livers than in maternal livers at two gestational stages, an average of 21% lower at day 14.5 (*P* = .04) and 40% lower at day 17.5 (*P* < .001). P50 values of fetal mouse red blood cells do not change substantially from day 12.5 to the end of gestation, showing only a slight increase just before birth (38), although the proportion of fetal hemoglobin synthesis decreases as gestation progresses (5,39). We observed no changes in AP50 between the two gestation stages.

Interestingly, placental AP50 decreased by approximately 18% (*P* = .003) from day 14.5 to day 17.5. In humans, a decrease in placental So_2_oxygen saturation has been shown, from 66.9% at 13–16 weeks to 52.1% at 37–40 weeks (6,40). Cordocentesis results showed reduced umbilical venous and arterial Po_2_partial pressure of oxygen levels with gestation and a decrease in placental intervillous Po_2_partial pressure of oxygen (41). This implies that fetal oxygen consumption increases considerably in near-term fetuses, because the maternal arterial Po_2_partial pressure of oxygen remains the same with gestation (42). The observed increase in placental oxygen affinity at the late gestation stage may be explained by this increase in fetal oxygen consumption.

Increased oxygen affinity of fetal hemoglobin is common among mammals (43). Results of several studies have suggested that this relationship is not only beneficial but essential for the survival and normal development of the fetus. Experimentally elevating the affinity of maternal hemoglobin in pregnant rats resulted in placental hypertrophy and smaller pups (44). The fetus can compensate for changes in oxygen delivery by altering oxygen affinity or extraction. Increased oxygen affinity of fetal hemoglobin oxygen has been shown in response to maternal smoking (45). Increased fetal hemoglobin synthesis has been reported in response to maternal hypoxia in baboons (46), lambs (47), and humans (48) and in response to prenatal complications, such as intrauterine growth restriction associated with placental insufficiency (49) and diabetes (50). These findings imply that fetal oxygen affinity reflects the well-being of the fetus, and that changes in affinity may serve as indicators of pathologic development in the fetus or in the placenta.

To test the applicability of this experimental setup in a clinically relevant setting, we applied two short protocols. Only small differences in mean AP50 values were found for the placenta and fetal liver compared with the full hyperoxia-to-hypoxia protocol, differences that may result from eliminating the hypoxic period, thus removing the left tail of the curves and shifting AP50 toward higher values.

Several limitations of this study are noted. The mean region of interest sizes of placental and fetal livers were significantly smaller compared with those in maternal livers (*P* < .0001), which may have resulted in less accuracy for tracing and marking them. However, both the placenta and fetal liver contain a large fraction of blood volume, producing high contrast on T2-weighted rapid acquisition with refocused echoes images, and therefore, were easily distinguished from their surroundings. R1 values do not depend solely on the change in dissolved oxygen, but may also reflect changes in arterial blood flow (51,52). This effect should be minimal for the three-dimensional gradient-echo sequence used in our study. BOLDblood oxygen level dependent contrast response to the oxygen inhalation challenge depends not only on deoxyhemoglobin, but also on physiologic changes in blood flow, vessel diameter, blood volume, and local changes in capillary hematocrit levels (14,15,53–55). Independent measurements of Po_2_partial pressure of oxygen and So_2_oxygen saturation were not feasible due to the small sizes of the mouse placental blood vessels and the invasiveness of the procedure. However, previous studies in sheep fetuses demonstrated a close correlation between changes in organ tissue oxygenation and changes in the BOLDblood oxygen level dependent MR imaging signal intensity as assessed with an implanted optode (19) and by fetal arterial blood sampling (18). Our studies were performed at 9.4 T and should be validated at lower-field MR imaging.

In conclusion, we present a noninvasive approach for obtaining MR imaging–based oxygen-hemoglobin dissociation curves and for deriving AP50 values and maps for the placenta and fetus. We believe that this approach could provide valuable information on the state of the fetus, both in preclinical animal models and also in clinical, prenatal monitoring of high-risk pregnancies.

**Advances in Knowledge**

■ A combination of two MR imaging contrast mechanisms, namely blood oxygen level–dependent T2* and oxygen-weighted T1, under a hyperoxia-to-hypoxia respiration challenge, enabled generation of oxygen-hemoglobin dissociation curves noninvasively in pregnant mice at embryonic days 14.5 (3 days after the formation of the placenta) and 17.5 (near term).
■ The apparent P50 (oxygen tension at which hemoglobin is 50% saturated) values were derived from the curves as a measure of oxygen-hemoglobin affinity; significantly lower mean apparent P50 values were shown in fetal livers than in maternal livers for both gestational stages (day 14.5: 21% ± 5 [*P* = .04] and day 17.5: 41% ± 7 [*P* < .0001]); the placenta displayed a reduction of 18% ± 4 in mean apparent P50 values from day 14.5 to day 17.5 (*P* = .003).
■ The applicability of this experimental setup was tested in a clinically relevant setting by using shorter protocols that excluded the hypoxic period and provided MR imaging–based oxygen-hemoglobin dissociation curves, with comparable mean apparent P50 values for the placenta and fetal liver compared with those of the full hyperoxia-to-hypoxia protocol.

**Implication for Patient Care**

■ Oxygen-hemoglobin affinity information may provide a marker of pathologic development in the fetus or in the placenta; therefore, in the future, once verified also at lower field MR imaging, a similar technique has the potential to be used for monitoring of high-risk pregnancies.

## APPENDIX

Appendix E1 (PDF)

## SUPPLEMENTAL FIGURES

Figure E1:

Figure E2:
