# Supplementary material for: MR Imaging–derived Oxygen-Hemoglobin Dissociation Curves and Fetal-Placental Oxygen-Hemoglobin Affinities
Source: Radiology. 2016 Jan 14;280(1):68–77. doi: 10.1148/radiol.2015150721 (PMC4942994; doi:10.1148/radiol.2015150721)
Supplement: Appendix E1 (PDF) [file ry150721suppa1.pdf]

©RSNA, 2016

10.1148/radiol.2016150721

Appendix E1

R1 values at each oxygen phase ($R1_t$) were extrapolated from the baseline by using the T1-weighted signal intensity (SI_0 and SI_t).

Derivation of R1 values using the variable flip angle method requires correction, as the flip angles can vary spatially across the image. This may lead to errors of up to 10% in the flip angle and, consequently, 20% errors in measured T1 values (56–58). To compensate for imperfections in flip angle distribution, we performed a set of calibration experiments. R1 values in maternal thigh muscle (n = 4 mice) were derived using a conventional saturation recovery sequence (5000, 2000, 1000, 800, 700, 600, 500, 400, 300, 200/10; section thickness, 1.0 mm; field of view, 5 × 5 cm^2; matrix, 128 × 64, zero filled to 128 × 128; averages, two), according to the following equation:

$$\mathrm{SI} = M_0\left(1 - Ae^{-\mathrm{TR}\cdot\mathrm{R1}}\right)$$

where SI is the measured signal intensity for each repetition time, M_0 is the steady-state signal intensity in fully relaxed images and A is approximately 1 for steady-state saturation.

The mean R1 was found to be 0.58 sec^{-1} ± 0.04. The correction factor for the flip angle was then calculated for each pregnant mouse from the deviation of R1 derived from the variable flip angle method compared with the mean R1.

References

56. Stollberger R, Wach P. Imaging of the active B1 field in vivo. Magn Reson Med 1996;35(2):246–251.

57. Clare S, Alecci M, Jezzard P. Compensating for B(1) inhomogeneity using active transmit power modulation. Magn Reson Imaging 2001;19(10):1349–1352.

58. Young IR, Bryant DJ, Payne JA. Variations in slice shape and absorption as artifacts in the determination of tissue parameters in NMR imaging. Magn Reson Med 1985;2(4):355–389.
