# Supplementary figures and images for: MR Imaging–derived Oxygen-Hemoglobin Dissociation Curves and Fetal-Placental Oxygen-Hemoglobin Affinities
Source: Radiology. 2016 Jan 14;280(1):68–77. doi: 10.1148/radiol.2015150721 (PMC4942994; doi:10.1148/radiol.2015150721)

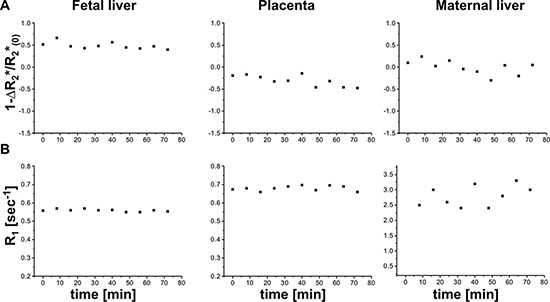

Supplement: Figure E1: [file rg150721suppf1.jpg]

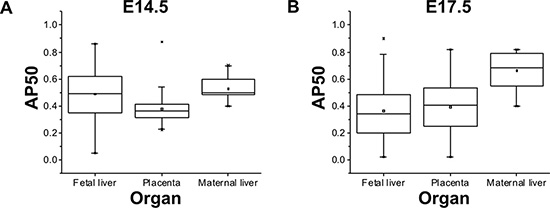

Supplement: Figure E2: [file rg150721suppf2.jpg]
